# Ultra-processed food consumption and risk of infertility in adults: a systematic review and meta-analysis

**DOI:** 10.3389/fpubh.2026.1906808

**Published:** 2026-08-12

**Authors:** Yanzhong Feng, Hui Miao, Na Li

**Affiliations:** 1 Department of Reproductive Genetics, Heping Hospital Affiliated to Changzhi Medical College, Changzhi, Shanxi, China; 2 Institute of Reproduction and Genetics of Changzhi Medical College, Changzhi, Shanxi, China; 3 Key Laboratory of Reproduction and Genetics of Changzhi Medical College, Changzhi, Shanxi, China; 4 Key Laboratory of Reproduction Engineer of Shanxi Health Committee, Changzhi, Shanxi, China

**Keywords:** meta-analysis, NOVA classification, reproductive health, risk of infertility, ultra-processed food

## Abstract

**Background:**

Ultra-processed foods (UPFs) have been linked to multiple adverse health outcomes; however, their association with infertility remains unclear. We conducted a systematic review and meta-analysis to evaluate the relationship between UPF consumption and infertility risk in adults.

**Methods:**

PubMed, Web of Science, and Scopus were searched from inception to May 31, 2026. Eligible studies assessed UPF intake using validated dietary assessment tools based on the NOVA classification and reported infertility outcomes among adults aged ≥18 years. Two reviewers independently conducted study selection, data extraction, and risk of bias assessment using the Newcastle–Ottawa Scale and the Joanna Briggs Institute checklist. Pooled relative risks (RRs) with 95% confidence intervals (CIs) were estimated using random-effects models. Heterogeneity was assessed using the *I^2^* statistic.

**Results:**

Four studies providing five independent effect estimates and including 8,769 participants (8,118 women and 651 men) were included. In the random-effects model, higher UPF consumption was not significantly associated with infertility risk (RR = 1.26, 95% CI: 0.94–1.69), although the point estimate suggested a positive direction of association. Substantial between-study heterogeneity was present (*I^2^* = 72.0%, *p* = 0.006). In sex-stratified analyses, no significant association was observed among women (RR = 1.26, 95% CI: 0.79–2.00; *I^2^* = 68.4%). Subgroup analyses by study design and exposure assessment yielded no statistically significant associations under random-effects models.

**Conclusion:**

Current evidence does not show a statistically robust association between UPF consumption and infertility under random-effects models, although estimates consistently suggest a positive trend. Evidence remains limited by few studies, substantial heterogeneity, and self-reported outcomes. Larger prospective, sex-specific studies are needed.

## 1 Introduction

Infertility is a major public health concern affecting 48 million couples and 186 million individuals worldwide (1). Defined as the failure to achieve pregnancy after 12 months or more of regular unprotected sexual intercourse, infertility affects approximately one in six people during their lifetime (2). Beyond its impact on reproductive outcomes, infertility is associated with substantial psychological distress, impaired quality of life, social stigma, and considerable economic burden due to fertility treatments and healthcare utilization (3). Given its increasing prevalence and multifactorial etiology, identifying modifiable risk factors for infertility has become a public health priority (4).

Lifestyle factors, including diet, have emerged as important determinants of reproductive health (5, 6). Previous studies have shown that adherence to healthy dietary patterns may improve fertility outcomes, whereas Western-style diets characterized by high intakes of refined grains, processed meats, saturated fats, and added sugars may adversely affect reproductive function (7). Growing evidence suggests that the degree of food processing may also play a critical role in shaping health outcomes (8, 9).

Ultra-processed foods (UPFs), as defined by the NOVA classification system, are industrial formulations manufactured using ingredients extracted from foods and cosmetic additives, with little or no intact whole-food content (10). Common examples include sugar-sweetened beverages, packaged snacks, confectionery products, instant meals, and processed meat products. UPFs are typically energy-dense and rich in added sugars, sodium, and unhealthy fats while being low in dietary fiber and essential nutrients (11). Over recent decades, UPF consumption has increased substantially worldwide and now contributes a large proportion of total energy intake in many populations (12–14).

High consumption of UPFs has been consistently associated with adverse health outcomes, including obesity, type 2 diabetes, cardiovascular disease, and metabolic syndrome (15, 16). Several biological mechanisms suggest that UPFs may also influence reproductive health. Excessive UPF intake may promote obesity, insulin resistance, chronic inflammation, oxidative stress, and hormonal dysregulation, all of which have been implicated in infertility (8, 17, 18). Furthermore, exposure to food additives and packaging-derived chemicals, such as bisphenols and phthalates, may interfere with endocrine function and reproductive processes (19–21).

An increasing number of epidemiological studies have examined the association between UPF consumption and fertility-related outcomes (8, 9, 22). Higher UPF intake has been linked to impaired semen quality, adverse reproductive health indicators, and reduced fecundability (23). More recently, observational studies have investigated the relationship between UPF consumption and infertility risk. However, the findings remain inconsistent, with some studies reporting positive associations (8, 22) and others showing null or inconclusive results (24, 25).

To date, the overall evidence regarding UPF consumption and infertility has not been comprehensively synthesized. Therefore, we conducted a systematic review and meta-analysis to evaluate the association between ultra-processed food consumption and infertility among adults. By quantitatively summarizing the available evidence, we aimed to provide a clearer understanding of whether higher UPF intake is associated with infertility risk.

## 2 Methods

### 2.1 Study design and eligibility criteria

This systematic review and meta-analysis was conducted in accordance with the Preferred Reporting Items for Systematic Reviews and Meta-Analyses (PRISMA 2020) guidelines (26). The study eligibility criteria were established *a priori* using the PECOS (Population, Exposure, Comparator, Outcome, and Study Design) framework. Reviews, editorials, commentaries, conference abstracts lacking sufficient quantitative data, case reports, animal studies, and *in vitro* studies were excluded.

Eligible studies included adults aged ≥18 years from either community-based or clinical populations. The exposure of interest was ultra-processed food (UPF) consumption, assessed using validated dietary assessment methods (e.g., food frequency questionnaires, dietary recalls, or dietary records) and classified according to the NOVA food classification system or an equivalent food-processing framework. The comparator group comprised participants with the lowest level of UPF consumption or the reference category defined by the original study. The primary outcome was infertility, defined as the inability to achieve pregnancy after at least 12 months of regular unprotected sexual intercourse, a physician- or clinically diagnosed infertility condition, or self-reported infertility according to the definitions adopted by the included studies.

### 2.2 Information sources and search strategy

A comprehensive literature search was conducted in PubMed, Web of Science, and Scopus from database inception to 31 May 2026. Search strategies combined controlled vocabulary terms and free-text keywords related to ultra-processed foods and infertility. The search strategy was tailored to the indexing system and search functionality of each database. To identify additional eligible studies, the reference lists of all included articles and relevant review papers were manually screened. No restrictions were imposed on geographic location. The complete search strategies for all databases are provided in Supplementary Table S1.

### 2.3 Study selection and data extraction

All retrieved records were imported into EndNote X9 (Clarivate Analytics, Philadelphia, PA, USA), and duplicate records were removed. Two reviewers (YF and HM) independently screened titles and abstracts for potential eligibility. Full-text articles of potentially relevant studies were subsequently assessed against the predefined inclusion and exclusion criteria. Discrepancies were resolved through discussion and consensus, with consultation from a third reviewer (NL) when necessary. The study selection process was documented using a PRISMA flow diagram.

Data extraction was independently performed by two reviewers using a standardized and piloted extraction form. The following information was collected from each study: First author and publication year; Country or region of study; Study design; Sample size and participant characteristics; Dietary assessment method; Definition and classification of UPF exposure; Infertility outcome definition and ascertainment method; Effect estimates with corresponding 95% confidence intervals (CIs); Variables included in multivariable-adjusted models. When multiple adjusted estimates were reported, the estimate with the most comprehensive adjustment for potential confounding factors was extracted. Any discrepancies in data extraction were resolved through discussion and consensus.

### 2.4 Risk of bias assessment

The methodological quality of included studies was independently evaluated by two reviewers. Cohort and case–control studies were assessed using the Newcastle–Ottawa Scale (NOS) (27), which evaluates methodological quality across three domains: selection of study participants, comparability of study groups, and ascertainment of exposure or outcomes. Cross-sectional studies were evaluated using the Joanna Briggs Institute (JBI) Critical Appraisal Checklist for Analytical Cross-Sectional Studies (28). Studies were categorized as having low, moderate, or high risk of bias according to established scoring criteria for the respective assessment tools. Disagreements were resolved through discussion and, when necessary, consultation with a third reviewer.

### 2.5 Statistical analysis

The association between UPF consumption and infertility risk was quantified using pooled effect estimates and corresponding 95% confidence intervals (CIs). Odds ratios (ORs), risk ratios (RRs), and hazard ratios (HRs) were extracted from individual studies. Because infertility is a relatively uncommon outcome in the general population, ORs and HRs were considered reasonable approximations of RRs and were pooled as equivalent measures of association. For studies reporting results across multiple categories of UPF consumption, the comparison between the highest and lowest exposure categories was used in the primary meta-analysis. Summary effect estimates were calculated using random-effects models based on the DerSimonian–Laird method (29), accounting for both within-study and between-study variability. Statistical heterogeneity was assessed using Cochran’s Q test and quantified using the *I^2^* statistic (30). *I^2^* values of approximately 25%, 50%, and 75% were interpreted as indicating low, moderate, and high heterogeneity, respectively. Subgroup analyses were conducted to explore potential sources of heterogeneity according to sex, study design, and exposure comparator. All analyses were performed using R software (version 4.3.1, R Foundation for Statistical Computing), and a two-sided *p* value <0.05 was considered statistically significant.

## 3 Results

### 3.1 Study selection

**Figure 1:**
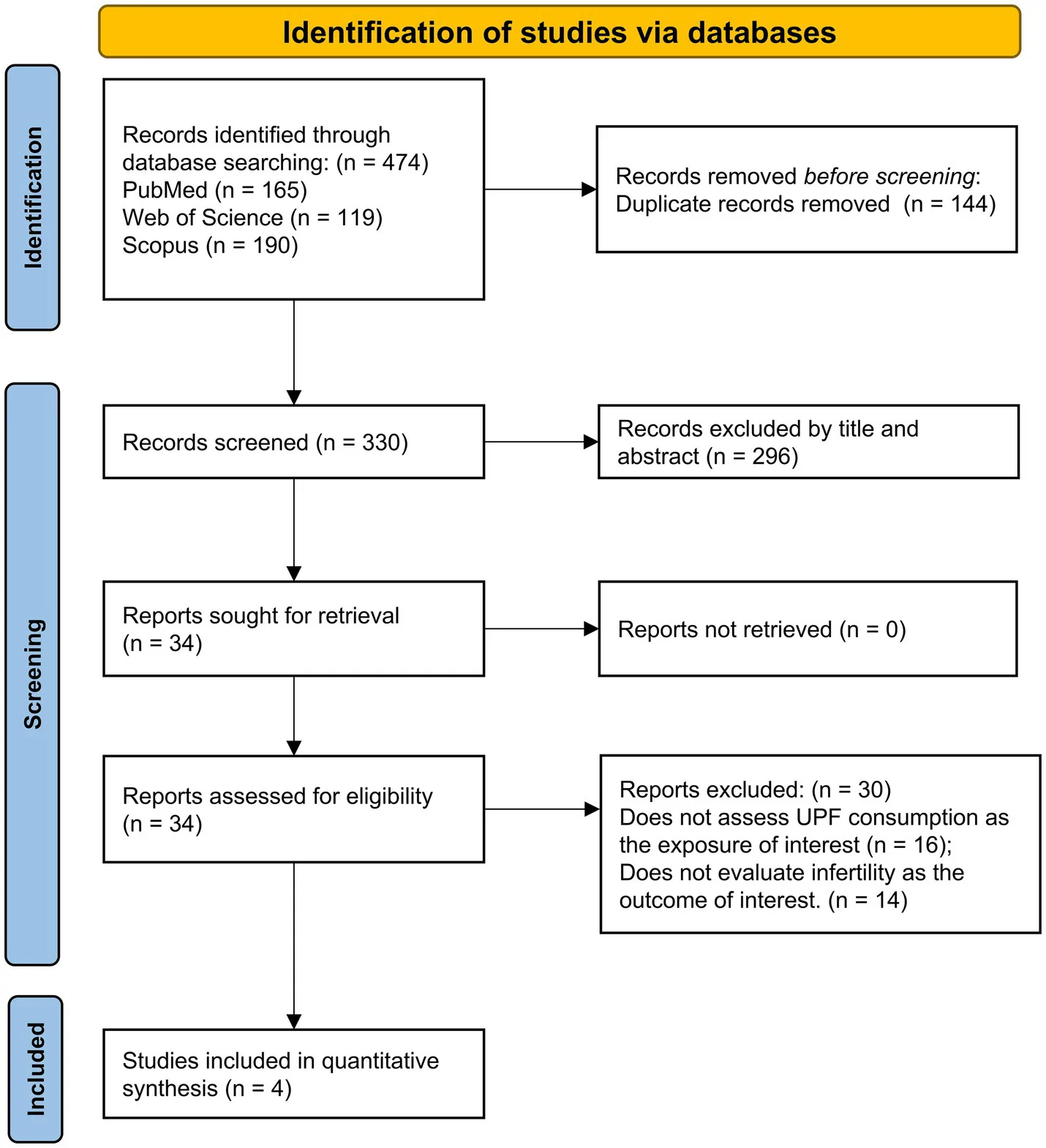
Study selection process for the systematic review and meta-analysis.

A total of 474 records were identified through database searching, including 165 from PubMed, 119 from Web of Science, and 190 from Scopus. After removal of 144 duplicates, 330 unique records remained for title and abstract screening. Of these, 296 were excluded as clearly irrelevant, leaving 34 articles for full-text review. All full-text reports were successfully retrieved and assessed for eligibility. Following full-text evaluation, 30 articles were excluded because they did not assess ultra-processed food (UPF) consumption as the exposure of interest and/or did not evaluate infertility as the outcome of interest. Ultimately, four studies fulfilled all predefined inclusion criteria and were included in the quantitative synthesis (meta-analysis) (8, 22, 24, 31). The detailed study selection process is presented in Figure 1.

### 3.2 Characteristics of included studies

Four studies comprising five independent effect estimates and 8,769 participants were included in the analysis (Table 1). Three studies were conducted in the United States and one in the Netherlands. Three studies employed a cross-sectional design, whereas one was a prospective cohort study that contributed separate estimates for female and male infertility. Overall, 8,118 women and 651 men aged 18–45 years were included. UPF intake was assessed using 24-h dietary recalls in three studies and a validated semi-quantitative food frequency questionnaire in one study. All studies classified UPFs according to the NOVA system (Group 4) and quantified exposure as the proportion of total daily food intake attributable to ultra-processed foods. Infertility was uniformly defined as failure to conceive after ≥12 months of attempting pregnancy and was assessed through self-report in all studies. All effect estimates were derived from multivariable-adjusted models accounting for key demographic, lifestyle, and reproductive characteristics, although the covariates included varied across studies.

**Table 1:** Characteristics of observational studies included in the meta-analysis examining the association between ultra-processed food intake and infertility risk.

Author (Year)	Country	Study design	Sample size	Participant characteristics	Dietary assessment	UPF classification	Exposure metric	Infertility definition	Infertility ascertainment	Effect estimate (95% CI)	Covariates adjusted
Lin et al. (2026) (24)	Netherlands	Prospective cohort	831 women; 651 men	Women mean 32.1 years; men mean 34.2 years	Dutch semi-quantitative HELIUS	NOVA Group 4	Percentage of total daily grams from UPFs	Attempting conception for ≥12 months without pregnancy	Self-reported questionnaires	Women: HR 1.01 (0.92–1.11) per standard deviation score (SDS) increase; Men: HR 1.36 (1.11–1.67) per SDS increase	Adjusted effect estimates accounted for maternal age, ethnicity, educational level, alcohol consumption, smoking status, pre-pregnancy BMI, parity, and total energy intake among women, and paternal age, ethnicity, educational level, alcohol consumption, smoking status, BMI, and total energy intake among men.
Baric et al. (2026) (31)	USA	Cross-sectional	2,582 women	Women aged 20–45 years	24-h dietary recall	NOVA Group 4	Percentage of total daily grams from UPFs	Attempting conception for ≥12 months without pregnancy	Self-reported questionnaires	OR 2.58 (1.10–5.88) per SDS increase	Age, race/ethnicity, poverty-to-income ratio, education, marital status, hormonal birth control use, other hormone use, history of pelvic infection, age at menarche, smoking status, physical activity, caloric intake, obesity status.
Su et al. (2025) (8)	USA	Cross-sectional	1,645 women	Women aged 18–44 years	24-h dietary recalls	NOVA Group 4	Percentage of total daily grams from UPFs	Attempting conception for ≥12 months without pregnancy	Self-reported questionnaires	OR 1.43 (95% CI 1.03–2.00) for highest vs. lowest quartile of UPF intake	Race, education, ratio of family income to poverty (RFIP), smoking, alcohol, physical activity
Evans et al. (2025) (22)	USA	Cross-sectional	3,060 women	Women aged 18–45 years	24-h dietary recalls	NOVA Group 4	Percentage of total daily grams from UPFs	Attempting conception for ≥12 months without pregnancy	Self-reported questionnaires	OR 1.26 (95% CI 0.91–1.74) for highest vs. lowest tertile of UPF intake	Age, marital status, income and BMI

### 3.3 Risk of bias assessment

The methodological quality of the included studies was generally high (Supplementary Tables S2, S3). The cohort study was judged to have a low risk of bias, achieving 8 of 9 stars on the Newcastle–Ottawa Scale. The three cross-sectional studies met most JBI quality criteria, with one study fulfilling all eight domains and the remaining two demonstrating only minor methodological limitations related to outcome ascertainment. Specifically, infertility was primarily assessed through self-report, introducing some uncertainty regarding the validity of outcome measurement. Nevertheless, all studies clearly defined participant selection criteria, employed appropriate methods for exposure assessment, adequately addressed key confounding factors, and used suitable statistical analyses. No study was at high risk of bias.

### 3.4 Meta-analysis

**Figure 2:**
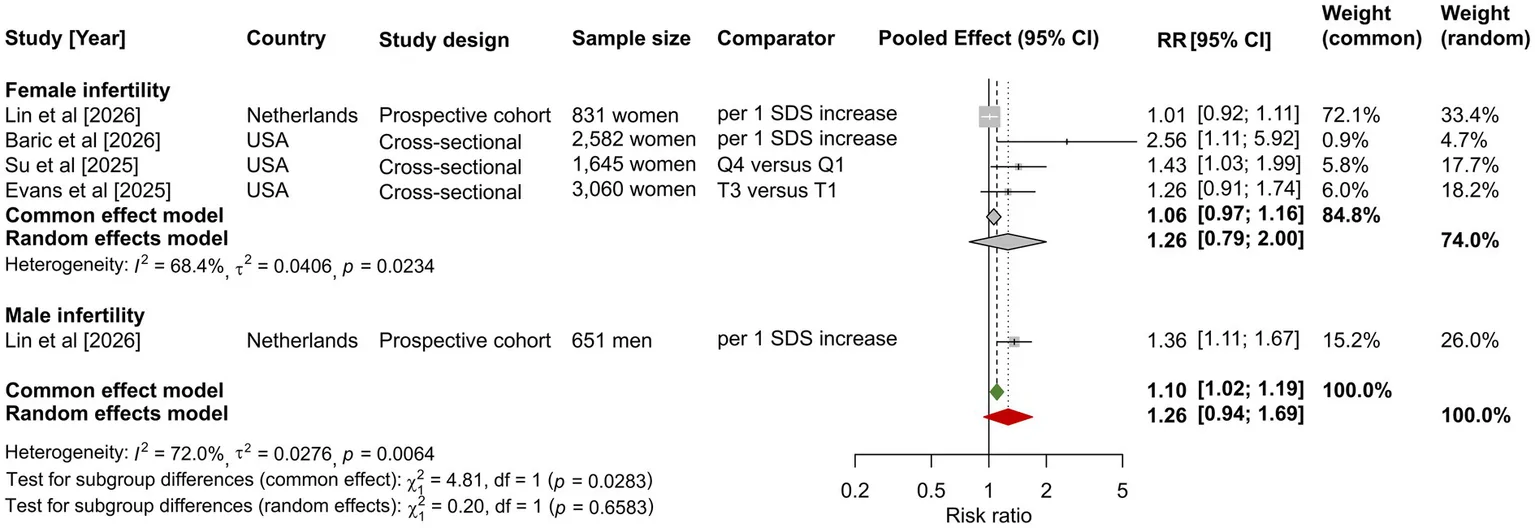
Forest plot of the association between UPF intake and infertility risk, stratified by sex. Heterogeneity within subgroups and overall was assessed using *I*^2^ and Cochran’s *Q*. SDS, standard deviation score; UPF, ultra-processed food. Per 1 SDS increase represents the effect associated with a one-standard-deviation increase in UPF exposure. Q4 versus Q1 denotes the highest versus lowest quartile of UPF intake, and T3 versus T1 denotes the highest versus lowest tertile of UPF intake.

Five effect estimates from four studies were included in the meta-analysis. The pooled estimate from the random-effects model suggested a potential positive association between UPF intake and infertility risk; however, the association did not reach statistical significance (RR = 1.26, 95% CI: 0.94–1.69). Substantial between-study heterogeneity was observed (*I^2^* = 72.0%, *p* = 0.006) (Figure 2). In sex-specific analyses, no significant association was observed among women (random-effects RR = 1.26, 95% CI: 0.79–2.00; *I^2^* = 68.4%), whereas the single study of men reported a significant positive association (RR = 1.36, 95% CI: 1.11–1.67; Figure 2).

**Figure 3:**
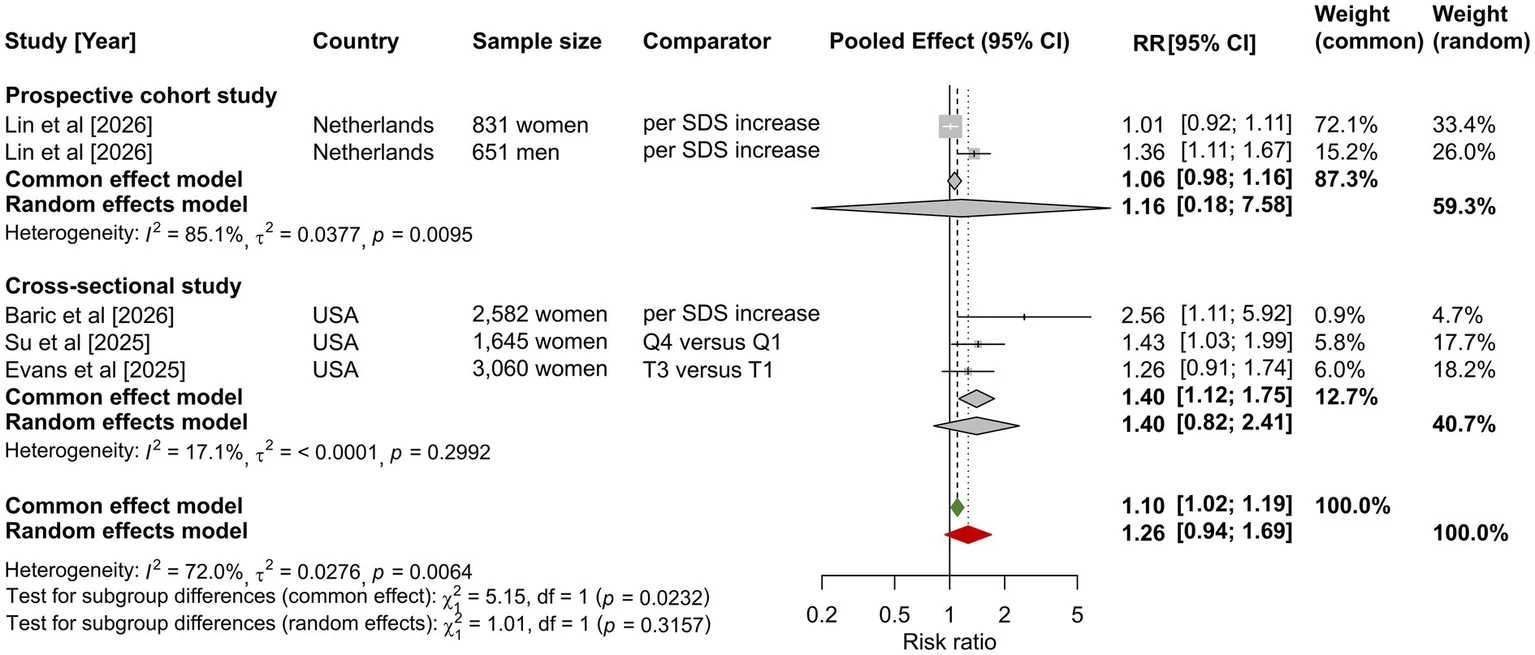
Forest plot of the association between UPF intake and infertility risk, stratified by study design. Heterogeneity within subgroups and overall was assessed using *I*^2^ and Cochran’s *Q*. SDS, standard deviation score; UPF, ultra-processed food. Per 1 SDS increase represents the effect associated with a one-standard-deviation increase in UPF exposure. Q4 versus Q1 denotes the highest versus lowest quartile of UPF intake, and T3 versus T1 denotes the highest versus lowest tertile of UPF intake.

**Figure 4:**
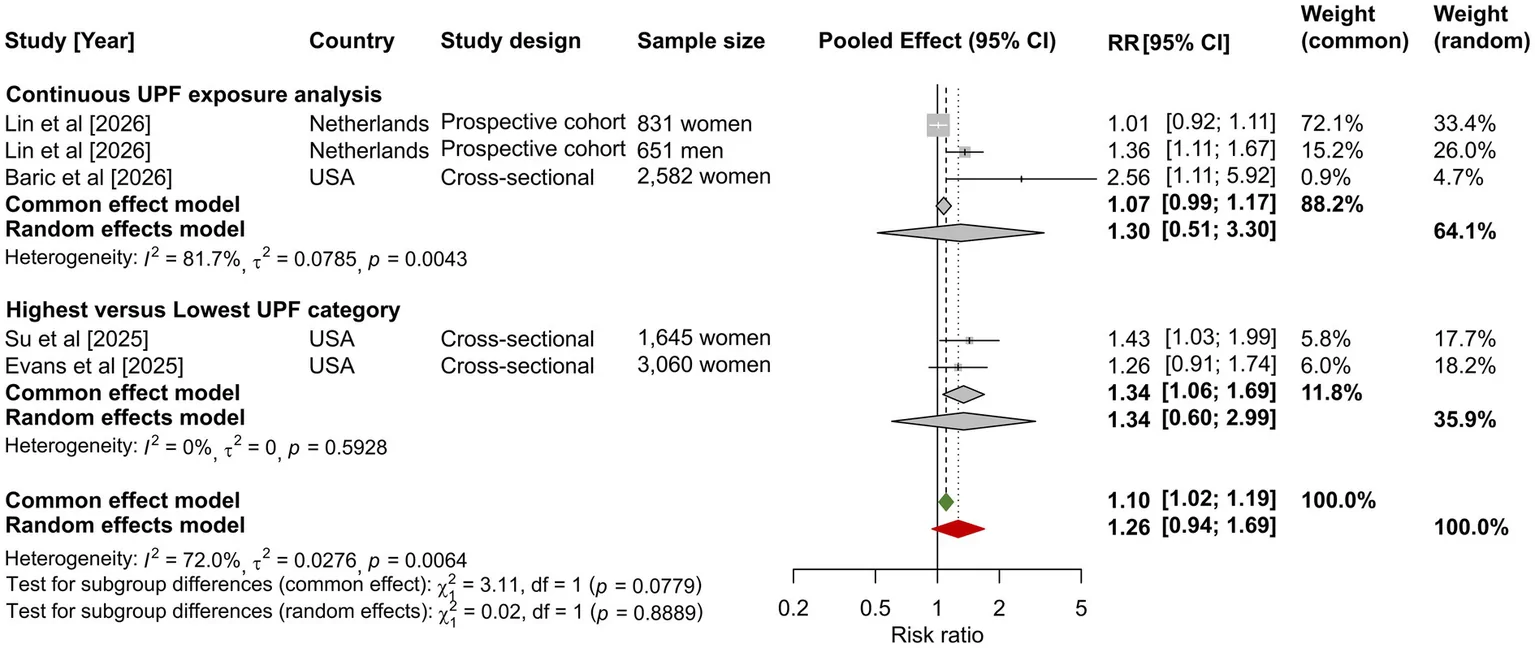
Forest plot of the association between UPF intake and infertility risk, stratified by exposure comparator. Heterogeneity within subgroups and overall was assessed using *I*2 and Cochran’s *Q*. SDS, standard deviation score; UPF, ultra-processed food. Per 1 SDS increase represents the effect associated with a one-standard-deviation increase in UPF exposure. Q4 versus Q1 denotes the highest versus lowest quartile of UPF intake, and T3 versus T1 denotes the highest versus lowest tertile of UPF intake.

By study design, neither cohort studies (random-effects RR = 1.16, 95% CI: 0.18–7.58; *I^2^* = 85.1%) nor cross-sectional studies (random-effects RR = 1.40, 95% CI: 0.82–2.41; *I^2^* = 17.1%) showed statistically significant associations (Figure 3). Similarly, analyses stratified by exposure metric found no significant associations under the random-effects model for either continuous UPF exposure (RR = 1.30, 95% CI: 0.51–3.30; *I^2^* = 81.7%) or highest-versus-lowest category comparisons (RR = 1.34, 95% CI: 0.60–2.99; *I^2^* = 0%; Figure 4). No significant subgroup differences were detected according to exposure metric (*p* = 0.889).

## 4 Discussion

This systematic review and meta-analysis represent the first comprehensive quantitative synthesis of evidence on the association between UPF consumption and infertility risk in adults. Drawing on four studies encompassing five independent effect estimates and 8,769 participants, our pooled analysis yielded a positive but non-statistically significant association between higher UPF intake and infertility under the random-effects model (RR = 1.26, 95% CI: 0.94–1.69), accompanied by substantial between-study heterogeneity (*I^2^* = 72.0%). Although the point estimate suggests a potentially clinically meaningful increase in risk, the wide confidence interval spanning unity and the high degree of heterogeneity preclude firm causal inferences.

The direction of our pooled estimate is consistent with a growing body of epidemiological literature linking poor dietary quality and food processing to adverse reproductive outcomes (32–34). High consumption of diets rich in refined carbohydrates, saturated fats, and red or processed meats—dietary patterns that overlap substantially with UPF-heavy diets—has been associated with reduced semen quality, irregular ovulatory cycles, and longer time-to-pregnancy in multiple prospective cohort studies (35–37). In the landmark Nurses’ Health Study II, a diet high in trans fats and animal protein was associated with an elevated risk of ovulatory infertility, while a diet emphasizing plant-based proteins, high-fat dairy, and low-glycaemic carbohydrates was protective (38–40). Our findings extend this literature by isolating the dimension of food processing as an independent axis of dietary exposure, independent of macronutrient composition per se.

Among the included studies, the single available estimate for male infertility—drawn from a well-powered prospective cohort—showed a statistically significant positive association (RR = 1.36, 95% CI: 1.11–1.67), whereas pooled estimates among women did not reach statistical significance (RR = 1.26, 95% CI: 0.79–2.00). However, this sex-specific divergence warrants careful interpretation. Three female estimates were derived from cross-sectional studies, which are inherently less suited to detecting prospective associations and may be subject to reverse-causation bias (41). Additionally, female infertility is etiologically heterogeneous—encompassing ovulatory disorders, tubal pathology, endometriosis, and diminished ovarian reserve—and cross-sectional study designs may lack the statistical power and temporal resolution to detect diet–outcome associations within these subgroups (42). Emerging evidence suggests that higher UPF intake may be associated with poorer semen quality, including adverse alterations in semen parameters (23, 43), providing additional support for potential biological pathways linking highly processed dietary patterns with male reproductive health. However, semen quality represents an intermediate reproductive phenotype and should be distinguished from clinically defined infertility (44). Therefore, while these findings strengthen the biological plausibility of a relationship between UPF exposure and reproductive dysfunction, additional prospective studies are required to determine whether UPF consumption contributes to increased infertility risk (45).

Several biological mechanisms have been proposed that could potentially link higher UPF consumption with impaired reproductive outcomes; however, these pathways were not directly evaluated in the included studies and should therefore be interpreted as hypotheses rather than explanations of the present findings. First, UPFs contribute to overweight and obesity, which are established risk factors for infertility (46). Excess adiposity can disrupt the hypothalamic–pituitary–gonadal axis, promote insulin resistance and hyperinsulinemia, and alter sex hormone metabolism, thereby impairing ovulation in women and spermatogenesis in men (47, 48). Second, UPFs are typically rich in added sugars, refined carbohydrates, saturated fats, and sodium, while being poor sources of dietary fiber, antioxidants, and essential micronutrients (49). This unfavorable nutritional profile may promote chronic low-grade inflammation and oxidative stress, both of which have been implicated in impaired oocyte quality, follicular development, and sperm DNA integrity (8, 50). Third, UPFs may increase exposure to food additives and endocrine-disrupting chemicals, including bisphenols and phthalates derived from food packaging (51). These compounds have been shown to interfere with steroidogenesis, reproductive hormone regulation, follicular development, and sperm function, potentially contributing to reduced fertility (52). Finally, UPF consumption has been associated with gut microbiome dysbiosis, increased intestinal permeability, and systemic inflammation (53). Alterations in the gut microbiota may also affect estrogen metabolism through the estrobolome, thereby influencing reproductive function (54). Nevertheless, these mechanisms remain hypothetical in the context of the present meta-analysis, as none of the included studies incorporated biomarker-based or mechanistic assessments. Given the limited number of available studies, substantial between-study heterogeneity, and the absence of a statistically significant association under the primary random-effects model, these potential pathways should be interpreted cautiously. Future prospective studies integrating repeated dietary assessments with biological measurements are needed to clarify whether these mechanisms mediate the relationship between UPF consumption and infertility risk.

Substantial heterogeneity was observed across studies (*I^2^* = 72.0%). Subgroup analyses according to sex, study design, and exposure metric did not meaningfully reduce heterogeneity, likely because the small number of included studies limited statistical power. Therefore, these analyses should be interpreted as exploratory and descriptive rather than definitive explanations of between-study variability. The observed heterogeneity may reflect multiple sources of methodological and population-level differences, including variations in study design, participant characteristics, dietary assessment methods, UPF exposure parameterization, infertility ascertainment, and covariate adjustment strategies. Specifically, the included studies differed in design (prospective cohort versus cross-sectional studies), study populations (women-only populations versus a mixed-sex cohort), and dietary assessment approaches (24-h dietary recalls versus semi-quantitative food frequency questionnaires). Although all studies classified UPFs according to the NOVA Group 4 system and used infertility definitions based on failure to conceive after ≥12 months of attempting pregnancy, differences in outcome ascertainment methods and analytical adjustment strategies may have introduced additional variability. Notably, NOVA classification is widely used to define UPFs, but its application has recognized limitations that may affect reproducibility and the interpretation of UPF exposure. First, NOVA classification is partly dependent on coder judgment, particularly when food descriptions are incomplete or products fall near category boundaries (55). Previous studies have reported only moderate inter-rater agreement and have used alternative classification scenarios to address uncertainty (56). Second, categorization can be challenging for mixed dishes, culturally specific foods, and borderline products, as processing levels may vary across ingredients and formulations (57). Moreover, differences in brands, reformulations, and available dietary information may result in inconsistent classification across studies (58). These limitations suggest that NOVA-based UPF assessment may involve measurement error and highlight the need for standardized, ingredient-level classification protocols to improve comparability and validity of findings (59). Collectively, these findings suggest that the observed heterogeneity likely represents genuine variation in exposure measurement, study populations, and analytical approaches rather than statistical artefacts alone (16). Future prospective studies using standardized infertility definitions, harmonized dietary assessment protocols, and comparable confounder adjustment strategies are warranted to better clarify the relationship between UPF consumption and infertility risk.

Despite its exploratory nature, these findings have preliminary public health and clinical relevance. Infertility represents a growing global health burden, with a substantial proportion of cases linked to modifiable lifestyle factors, including diet. If confirmed in prospective studies, an association between UPF consumption and infertility would support the integration of dietary counselling into preconception and fertility care. This is particularly relevant given the high intake of UPFs among reproductive-aged adults and their disproportionate consumption in socioeconomically disadvantaged groups, suggesting potential benefits for both reproductive and cardiometabolic health equity (60). In clinical practice, guidance on reducing UPF-dominant dietary patterns may be considered alongside established lifestyle recommendations such as weight management, physical activity, and avoidance of alcohol and tobacco (61).

Infertility is a multifactorial condition resulting from complex interactions among nutritional, metabolic, endocrine, inflammatory, environmental, and genetic factors. In addition to dietary modification, increasing evidence supports the potential role of nutraceutical interventions, including inositol supplementation and other targeted nutritional strategies, particularly among women with metabolic disorders or obesity, in improving reproductive outcomes (62, 63). Furthermore, chronic inflammation and immune dysregulation have emerged as important contributors to both female and male infertility (45), highlighting the importance of integrated approaches that combine dietary, lifestyle, and pharmacological interventions. Recent advances in the management of endometriosis and the understanding of epigenetic alterations associated with reproductive diseases further emphasize the complex biological pathways underlying infertility (64–66). These developments provide important clinical context and underscore the need for future prospective studies evaluating how dietary exposures, including UPF consumption, interact with metabolic, inflammatory, and reproductive pathways to influence fertility.

### 4.1 Limitation

Several limitations should be acknowledged. The small number of included studies limits statistical power, prevents robust subgroup analyses, and precludes formal assessment of publication bias, indicating that the findings should be considered exploratory. All studies relied on self-reported infertility, which may introduce misclassification and bias due to differences in understanding of the clinical definition and recall. In addition, the predominance of cross-sectional designs in women limits causal inference and raises concerns about reverse causation. Finally, the included studies were restricted to high-income countries (the United States and the Netherlands), limiting generalizability to other settings with different dietary patterns and UPF exposure levels. Future research should prioritize large prospective cohort studies to establish temporality and strengthen causal inference, ideally incorporating repeated dietary assessments and biomarker-based measures of reproductive function and related pathways. Clinically confirmed infertility outcomes and standardized measures such as time-to-pregnancy should be preferred over self-report. Sex-stratified analyses are needed to clarify potential differences between male and female reproductive outcomes, along with studies examining effect modification by metabolic and lifestyle factors. Mechanistic research on specific UPF components and randomized dietary interventions replacing UPFs with minimally processed foods would further advance causal understanding and inform targeted prevention strategies.

## 5 Conclusion

In summary, current evidence is insufficient to establish a consistent association between ultra-processed food consumption and infertility risk. Although pooled estimates indicate a potential increase in risk, the interpretation is limited by the small number of available studies, substantial between-study heterogeneity, and reliance on observational designs. Further well-designed prospective studies with standardized dietary assessment and clinically validated reproductive outcomes are needed to clarify the relationship between UPF consumption and infertility.

## Data Availability

The raw data supporting the conclusions of this article will be made available by the authors, without undue reservation.
