# Supplementary material for: Ultra-processed food consumption and risk of infertility in adults: a systematic review and meta-analysis
Source: Front Public Health. 2026 Aug 12;14:1906808. doi: 10.3389/fpubh.2026.1906808 (PMC13506803; doi:10.3389/fpubh.2026.1906808)
Supplement: Supplementary file 1 [file Data_Sheet_1.PDF]

Supplementary Table S1. Literature Search Strategy

Databases	Search terms
PubMed	(("Food, Processed"[Mesh] OR "ultra-processed food*"[tiab] OR "ultra processed food*"[tiab] OR "UPF"[tiab] OR "UPFs"[tiab] OR "processed food*"[tiab] OR "highly processed food*"[tiab] OR "industrial food*"[tiab] OR "industrialized food*"[tiab] OR "packaged food*"[tiab] OR "ready-to-eat food*"[tiab] OR "ready to eat food*"[tiab] OR "fast food*"[tiab] OR "junk food*"[tiab] OR "convenience food*"[tiab] OR "instant food*"[tiab] OR "NOVA classification"[tiab] OR "NOVA system"[tiab] OR "NOVA group 4"[tiab] OR "processed diet*"[tiab] OR "ultra-processing"[tiab] OR "ultra processing"[tiab])) AND (("Infertility"[Mesh] OR "Infertility, Female"[Mesh] OR "Infertility, Male"[Mesh] OR infertil*[tiab] OR subfertil*[tiab] OR fertility[tiab] OR fecundity[tiab] OR "reproductive health"[tiab] OR "reproductive outcome*"[tiab] OR "reproductive function"[tiab] OR "fertility status"[tiab] OR "time to pregnancy"[tiab] OR "pregnancy rate"[tiab] OR "live birth"[tiab] OR "clinical pregnancy"[tiab] OR "assisted reproduction"[tiab] OR "assisted reproductive technolog*"[tiab] OR ART[tiab] OR IVF[tiab] OR ICSI[tiab] OR "in vitro fertilization"[tiab] OR "intracytoplasmic sperm injection"[tiab] OR "semen quality"[tiab] OR "sperm quality"[tiab] OR "sperm concentration"[tiab] OR "sperm motility"[tiab] OR "sperm morphology"[tiab] OR "sperm DNA fragmentation"[tiab] OR "ovarian reserve"[tiab] OR AMH[tiab] OR AFC[tiab] OR "anti-mullerian hormone"[tiab] OR "anti-müllerian hormone"[tiab] OR ovulation[tiab] OR "ovulatory infertility"[tiab]))
Web of Science	TS= ("ultra-processed food*" OR "ultra processed food*" OR UPF OR UPFs OR "NOVA classification" OR "NOVA system" OR "industrial food*" OR "processed dietary pattern*" OR "western dietary pattern*" OR "highly processed food*" OR "fast food*" OR "processed meat*" OR "ready-to-eat food*" OR "packaged food*") AND TS= (infertility OR infertile OR subfertility OR subfertile OR fertility OR fecundity OR fecundability OR "time to pregnancy" OR "reproductive health" OR "reproductive outcome*" OR "ovulatory disorder*" OR "male fertility" OR "female fertility" OR "semen quality" OR "sperm quality")
Scopus	TITLE-ABS-KEY ("ultra-processed food*" OR "ultra processed food*" OR UPF OR UPFs OR "NOVA classification" OR "NOVA system" OR "industrial food*" OR "processed dietary pattern*" OR "western dietary pattern*" OR "highly processed food*" OR "fast food*" OR "processed meat*" OR "ready-to-eat food*" OR "packaged food*") AND TITLE-ABS-KEY (infertility OR infertile OR subfertility OR subfertile OR fertility OR fecundity OR fecundability OR "time to pregnancy" OR "reproductive health" OR "reproductive outcome*" OR "ovulatory disorder*" OR "male fertility" OR "female fertility" OR "semen quality" OR "sperm quality")

Supplementary Table S2. Quality assessment of included cohort studies by Newcastle-Ottawa Scale

		Selection				Comparability		Outcome			
Author	Year	Representativeness of exposed cohort	Selection of non-exposed cohort	Exposure Ascertainment	Outcome present at start of study	Study controls for age	Study controls for any additional important factor	Assessment of Outcome	Length of follow-up	Adequacy of follow-up	Score
Brinton LA, et al	2005	★	★	★	★	★	★	★	★	0	8

Cohort Studies: **Selection** ①Representativeness of the exposed cohort★②Selection of the non-exposed cohort★ ③Ascertainment of exposure★ ④Demonstration that outcome of interest was not present at start of study★. **Comparability** ①Comparability of cohorts on the basis of the design or analysis★★. **Outcome** ①Assessment of outcome★ ②Was follow-up long enough for outcomes to occur★ ③Adequacy of follow up of cohorts★.

Supplementary Table S3. Quality assessment of included cross-sectional studies by JBI Critical Appraisal Checklist

Author	Year	Were the criteria for inclusion in the sample clearly defined?	Were the study subjects and the setting described in detail?	Was the exposure measured in a valid and reliable way?	Were objective, standard criteria used for measurement of the condition?	Were confounding factors identified?	Were strategies to deal with confounding factors stated?	Were the outcomes measured in a valid and reliable way?	Was appropriate statistical analysis used?	Methodological quality
Baric et al	2026	Yes	Yes	Yes	No	Yes	Yes	Unclear	Yes	Moderate to High
Su et al	2025	Yes	Yes	Yes	Yes	Yes	Yes	Unclear	Yes	Moderate to High
Evans et al	2025	Yes	Yes	Yes	Yes	Yes	Yes	Yes	Yes	High
